# A Cost-Consequences analysis of the effect of Pregabalin in the treatment of peripheral Neuropathic Pain in routine medical practice in Primary Care settings

**DOI:** 10.1186/1471-2377-11-7

**Published:** 2011-01-20

**Authors:** Ana Navarro, María T Saldaña, Concepción Pérez, Sandra Torrades, Javier Rejas

**Affiliations:** 1 Primary Care Health Centre Puerta del Ángel, Madrid, Spain; 2 Primary Care Health Centre Raíces, Castrillón, Asturias, Spain; 3 Pain Clinics, Hospital de la Princesa, Madrid, Spain; 4 Department of Project Management, European Biometrics Institute, Barcelona, Spain; 5 Health Outcomes Research Department, Medical Unit, Pfizer España, Alcobendas, Spain

## Abstract

**Background:**

Neuropathic pain (NeP) is a common symptom of a group of a variety of conditions, including diabetic neuropathy, trigeminal neuralgia, or postherpetic neuralgia. Prevalence of NeP has been estimated to range between 5-7.5%, and produces up to 25% of pain clinics consultations. Due to its severity, chronic evolution, and associated co-morbidities, NeP has an important individual and social impact. The objective was to analyze the effect of pregabalin (PGB) on pain alleviation and longitudinal health and non-health resources utilization and derived costs in peripheral refractory NeP in routine medical practice in primary care settings (PCS) in Spain.

**Methods:**

Subjects from PCS were older than 18 years, with peripheral NeP (diabetic neuropathy, post-herpetic neuralgia or trigeminal neuralgia), refractory to at least one previous analgesic, and included in a prospective, real world, and 12-week two-visit cost-of-illness study. Measurement of resources utilization included both direct healthcare and indirect expenditures. Pain severity was measured by the Short Form-McGill Pain Questionnaire (SF-MPQ).

**Results:**

One-thousand-three-hundred-fifty-four PGB-naive patients [58.8% women, 59.5 (12.7) years old] were found eligible for this secondary analysis: 598 (44%) switched from previous therapy to PGB given in monotherapy (PGBm), 589 (44%) received PGB as add-on therapy (PGB add-on), and 167 (12%) patients changed previous treatments to others different than PGB (non-PGB). Reductions of pain severity were higher in both PGBm and PGB add-on groups (54% and 51%, respectively) than in non-PGB group (34%), p < 0.001. Incremental drug costs, particularly in PGB subgroups [€34.6 (80.3), €160.7 (123.9) and €154.5 (133.0), for non-PGB, PGBm and PGBadd-on, respectively (p < 0.001)], were off-set by higher significant reductions in all other components of health costs yielding to a greater total cost reductions: -€1,045.3 (1,989.6),-€1,312.9 (1,543.0), and -€1,565.5 (2,004.1), for the three groups respectively (p = 0.03).

**Conclusion:**

In Spanish primary care settings, PGB given either add-on or in monotherapy in routine medical practice was associated with pain alleviation leading to significant longitudinal reductions in resource use and total costs during the 12-week period of the study compared with non-PGB-therapy of patients with chronic NeP of peripheral origin. The use of non-appropriate analgesic therapies for neuropathic pain in a portion of subjects in non-PGB group could explain partially such findings.

## Background

Neuropathic pain (NeP), defined as a pain initiated or caused by a primary injury or malfunction of the nervous system, is a common symptom of a group of a variety of conditions, including diabetic neuropathy, trigeminal neuralgia, or postherpetic neuralgia [1,2]. Prevalence of NeP has been estimated to range between 5-7.5%, and produces up to 25% of pain clinics visits [3,4]. Due to its severity, chronic evolution, and associated comorbidities, NeP has an important individual and social impact, mainly due to the degree of disability when compared to patients with chronic pain of non-neuropathic origin [5]. NeP is associated with depression, anxiety disorders, and impairment of sleep quality. All this translates into a loss of quality of life affecting the family of patients, and the social and working environment [6-9]. Many patients with NeP are not properly diagnosed, do not receive the appropriate therapy, or are prescribed doses of appropriate drug lower than recommended [10,11]. All of this influences the burden of disease [12-14], and causing important healthcare and indirect costs [11,15-17].

The results of a systematic review suggested that anticonvulsive drugs, such as gabapentin or pregabalin, might be considered first-line treatments for peripheral NeP [18]. Pregabalin (PGB) is an alpha_2_-delta ligand of voltage gated calcium channels that displays analgesic, anxiolytic, and anticonvulsive properties [19]. In randomized, placebo-controlled clinical trials, PGB demonstrated its efficacy for pain relief in patients with diabetic neuropathy and peripheral postherpetic neuralgia, significantly improving mood symptoms, sleep, and quality of life [20-23]. However, the use of drugs in clinical trials markedly differs from that of the routine clinical practice in several aspects, thus limiting the generalization of results [24]. In this sense, real world, non-interventional studies may provide complementary information on the effectiveness of specific treatments in real clinical practice settings [25].

The objective of this research was to analyze the clinical and economic profile of PGB in a "Real World" setting in Spain. Observed data from the clinical practice are shown, indicating the effect of PGB on pain and its associated costs derived from the use of different health and non-health care resources in a group of patients with NeP of peripheral origin treated in routine clinical practice conditions for 12-weeks in Primary Care Settings (PCS).

## Methods

### Study Design

The results of a secondary analysis of a multicentre, observational and prospective 12-week study (LIDO study)[26] are presented. The LIDO study was designed with the objective of determining the prospective cost-of-illness of treating refractory NeP patients in real life conditions in PC settings in Spain. Also, patient's health status, disability, quality of life, sleep disturbances and symptoms associated with neuropathic pain in addition to pain measurements were assessed at baseline and after 12-weeks of follow-up. The study was carried out between September 2005 and April 2006, and 391 PCPs representative of the entire Spanish territory participated. Due to the non-interventional design of the study, only two visits (baseline and 12-weeks visit) where scheduled within the frame of the study. The analgesic treatment prescribed was determined by the clinical judgment of physicians, as the protocol did not establish any particular therapy. Doctors could substitute the previous treatment by one or several other drugs, or add a new drug to the existing therapy as duly appropriated. Study sampling and patients' selection requirements are described below in this section. In the LIDO study, healthcare resources utilization, along with the number of sick leaves and productivity while working in active population and patient-reported-outcomes variables were collected for a 3-months time frame and are described below (Additional file 1). The study was approved by the Ethics Committee of Clinical Research of the Hospital de la Princesa (Madrid).

The objective of this secondary analysis was to compare the effect on pain alleviation of two PGB regimes; add-on and monotherapy (PGB add-on and PGB monotherapy groups), with a therapeutic regime for NeP not including PGB (non-PGB group). The impact of such therapies on healthcare and non healthcare resources utilization and its corresponding costs was also analyzed.

### Study Population

Sampling in the original study [26] was carried out by means of a stratified multistage probabilistic sample without replacement. The sampling frame was all health regions from the 17 autonomous communities of Spain. The first stage consisted of the selection of the PCS within each health region. The number of PCS to be selected in each region was proportional to the population of the region. The PCS list was obtained from the catalogue of health centres of the Spanish Ministry of Health and the density of population from the National Institute of Statistics. The probability of selection of each clinic was related to the population of the area covered by the setting. A random process was applied to chose centers in each region. In the second stage, the center was contacted by phone in order to get a list of possible investigators considerd candidates for participation in the study. Then, a family physician or general practitioner per setting chosen at random within those with previous experience in clinical and epidemiological research was invited to participate. Those refusing to participate were replaced by others also selected at random in the same setting. The third stage consisted of the selection of patients. Every physician was responsible for selecting patients for the study, and was told to choose consecutive subjects (systematic sampling strategy) from the daily list of all patients with an appointment with each of the participating physician meeting inclusion and exclusion criteria mentioned below.

The study included men and women over 18 years with NeP secondary to diabetic neuropathy, postherpetic neuralgia, or trigeminal neuralgia according to ICD-10 codes. The subjects were refractory to previous analgesic therapy, and suffered chronic pain for at least 6 months. The term refractory was defined as the absence of pain reduction after treatment with at least one course of an analgesic drug in monotherapy. Despite the observational design of the original study [26], additional requirements for the secondary analysis included a NeP diagnostic questionnaire DN4 (Douleur Neuropathique 4 questions, range 0-10) scoring greater than or equal to four, which was interpreted as existing a neuropathic component in the pain [27], a sufficient cultural and educational level of patients to complete health questionnaires written in Spanish, and patient's informed consent. The secondary analysis included those patients fulfilling the previously mentioned selection criteria only, and who had not received PGB treatment before study initiation.

### Description of study variables

To avoid modifying the physician's common clinical practice, a baseline visit (week 0) and a final visit (week 12) were performed. In the baseline visit, selection criteria were checked and social-demographic variables collected, as well as disease duration, treatment, and use of health and non-healthcare resources in the 12 weeks prior to patient inclusion. Patients were requested to complete the scale DN4 at baseline visit only. At baseline and final visits, patients completed the SF-MPQ [28], and at each week patients recorded pain intensity in a diary (SF-MPQ visual analogue scale or VAS) (Additional file 1). As the original study was designed as an observational epidemiological research, forced adverse events reporting could not be implemented except to document the reason for discontinuation before the end of study visit.

### Use of healthcare resources and labour productivity

Information regarding healthcare resources used in last 12 weeks (pharmacological and non-pharmacological treatments, medical visits, hospitalizations, and complementary tests carried out due to pain) were obtained from the own patient and medical records in the two study visits (Additional file 1). Also, patients were interviewed on the impact of pain on their productivity at work during the last 12 weeks, and information was collected relative to the number of days patients did not work due to pain, days working with pain, and the self-perceived labour productivity they had on average on these occasions (determined as 0% to 100% productivity). From these data, calculations were made on the number of Lost-Workdays Equivalents (LWDE), through the application of the following formula: LWDE = W1 + W2 (1- P); where W1 is the number of days unable to work or conduct their daily activities due to pain in the last 12 weeks; W2 the number of days working with pain in the same period, (1-P) the percentage of labour disability at work, and P the percentage of effectiveness at work [29-31].

### Estimation of costs

Calculation of the total costs per patient included direct healthcare costs and indirect costs derived from LWDE. Drugs costs were obtained from the Pharmacists Catalogue from year 2006 [32], matching the public selling prices + VAT of the cheapest generic medications, or cheapest pharmaceutical specialties in case of unavailability of a generic medication. Costs of non-pharmacological treatments, medical visits, hospitalizations, and complementary tests were obtained from the Soikos healthcare costs database for year 2005, updated to year 2006 in agreement with the Consumption Prices Index, December 2005 (Table 1). Finally, the human capital method was applied to determine the cost of LWDE, and total national average wages per worker and month (first quarter 2006) divided by 30 days were obtained from the National Institute of Statistics.

**Table 1:** Unitary costs of health resources and productivity losses.

Resource	Unitary cost (€)
Non-pharmacological treatment **(per session)**	
Physiotherapy	9.96
TENS	22.95
Infiltrations (ex. joint...)	146.34
Electrotherapy	7.36
Blockade (ex. epidural)	87.83
Iontophoresis	9.91
Spinal stimulator	6886.21
Pumps	8212.71
Hydrotherapy	5.91
Microwaves	6.24
Magnetotherapy	4.87
Acupuncture	35.00
**Medical visits**	
Primary care medical visit	19.81
Pain unit medical visit	51.23
Specialist medical visit	56.41
Emergency room medical visit	111.89
Hospitalization (one day)	300.52
**Complementary tests**	
CT	145.28
Resonance	343.66
Electromyogram	126.51
ECHO-Doppler	131.18
Thermogram	133.27
X-Rays	17.26
General analysis	23.48
Bone gammagram	133.25
**Productivity**	
Cost per lost-workday	51.27

TENS: Transcutaneous electric neurostimulation; CT: Computerized tomography

### Statistical Analysis

Statistical analysis was performed by European Biometric Institute (EBI), at Barcelona, an independent body engaged by the sponsor of the study. For statistical analyses, patients where classified into three groups: patients to whom one or several drugs other than PGB were substituted or added to the previous treatment (non-PGB group); patients to whom monotherapy with PGB was prescribed as a substitute of a previous therapy (PGB monotherapy group); and patients to whom PGB was added to the previous therapeutic schedule (PGB add-on group).

Descriptive statistics were calculated and the Kolmogorov-Smirnov test was used to verify the normal distribution. Analysis of variance (ANOVA), Kruskall-Wallis tests, and Chi^2 ^tests were used to check the homogeneity of baseline variables among groups. Responder rate (percentage of subjects showing a decline in baseline pain intensity equal or higher than 50% at the final visit) and weekly changes respect to baseline for McGill pain scale scores were used to analyze therapy effect on clinical consequences. Also, calculations were made of the cumulative number of days with no or mild pain (<40 mm in the SF-MPQ VAS). Absolute values were obtained to quantify the use of healthcare resources, LWDEs, and overall costs in the 12 weeks previous to each visit. Analyses of covariance (ANCOVA), adjusted with baseline values and number of previous drugs, were used to run between groups comparisons of the change of quantitative variables at ending visit. All analyses were conducted with the patients who completed the 12-week follow-up, for which the change from baseline could be calculated. Statistically significant of comparisons between groups were adjusted using the method of Tukey for multiple comparisons.

All statistical tests were two-sided and considered significant when attained a <0.05 p level. The SAS statistical package, version 8.2 was used for all statistical analyses.

## Results

### Patients Disposition

**Figure 1:**
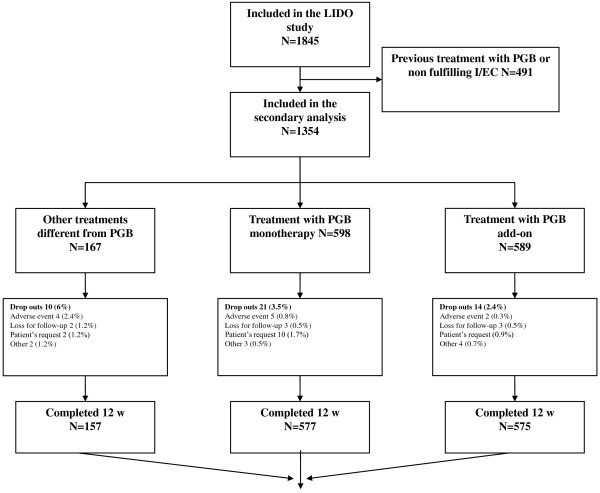
**Patients Disposition**. PGB: pregabalin I/EC: Inclusion/exclusion criteria.

A total of 1,845 patients were included in the original study, 1,354 of which had not been previously exposed to PGB. The analysis was conducted on 1,309 patients (96.7%) who completed 12 weeks of study. Forty-five patients withdrew due to: 11 (0.8%) adverse events, 8 (0.6%) lost to follow-up, 17 (1.3%) patients' decision, and 9 (0.7%) other causes (Figure 1). The most frequent cause of NeP was diabetic neuropathy (54.4%), followed by postherpetic neuralgia (33.8%), and trigeminal neuralgia (11.8%), without differences between study groups (table 2).

**Table 2:** Demographic and Clinical Characteristics

Characteristic	Non-PGB **(N = 157)**^**†**^	PGB monotherapy **(N = 577)**^**†**^	PGB add-on **(N = 575)**^**†**^	*** P***^**‡**^
Gender (female), n (%)	87 (55.3)	349 (60.4)	332 (57.8)	0.4874
Age, mean (SD)	60.4 (12.4)	58.6 (12.5)	59.7 (13.0)	0.1486
Body Mass Index (Kg/m^2^)	26.9 (3,5)	27.3 (3,8)	27.3 (3,9)	0.4673
Civil status (married or with couple), n (%)	108 (69.0%)	383 (66.4%)	389 (67.6%)	0.6041
Working status, n (%)				
Active	51 (32.5)	200 (34.6)	178 (31.0)	0.3835
Housewife	29 (18.7)	73 (12.7)	86 (14.9)	
Off sick	9 (6.0)	44 (7.6)	61 (10.6)	
Unemployed	3 (1.8)	15 (2.6)	11 (1.9)	
Retired	56 (35.5)	204 (35.3)	213 (37.0)	
Unknown	8 (5.4)	41 (7.1)	26 (4.6)	
Elapsed time from diagnosis (years), mean (SD)	2.1 (3.1)	1.9 (3.4)	2.0 (3.4)	0.7130
Diagnosis (%)				
Diabetic neuropathy	47.9	58.2	53.0	0.2852
Post-herpetic neuralgia	37.2	31.2	36.9	
Trigeminal neuralgia	14.9	10.7	10.1	
DN4 Questionnaire, mean (SD)	6.4 (1.7)	6.8 (1.8)	6.8 (1.7)	0.033
SF-MPQ, mean (SD)				
Total	18.5 (8.0)	21.0 (8.4)	21.1 (8.4)	0.002
PPI (0 - 5)	2.4 (0.9)	2.7 (0.8)	2.7 (0.9)	<0.001
VAS (0 - 100)	66.8 (17.6)	71.4 (15.2)	72.6 (15.7)	<0.001
Previous treatments^#^, n (%)				
Mean (SD)	2.4 (1.3)	2.0 (1.1)	2.6 (1.4)	<0.001
NSAID^$^	66 (42.0)	140 (24.3)	311 (54.1)	<0.001
Paracetamol	69 (43.7)	224 (38.8)	266 (46.3)	0.030
Metamizol	40 (25.7)	119 (20.7)	168 (29.2)	0.003
Opiates	70 (44.3)	153 (26.6)	244 (42.4)	<0.001
AED	43 (27.5)	128 (22.2)	108 (18.8)	0.043
Tricyclic drugs	17 (10.8)	59 (10.2)	67 (11.7)	0.704
Others^§^	12 (7.8)	35 (6.0)	79 (13.8)	<0.001

SD: Standard deviation; NSAID: non-steroidal anti-inflammatory drug; AED: antiepileptic drug; PGB: pregabalin; VAS: visual analogue scale; PPI: present pain intensity; SF-MPQ: Short Form McGill Pain Questionnaire; DN4: Doleur Neuropathique 4 questions scale. ^†^Total number of analyzed patients; some patients did not report all data; ^**‡**^Between groups comparison; ^#^Patients might be receiving more than one previous treatment; ^$^Other than metamizol; ^§^Includes vitamins, benzodiazepines, fosfosal, duloxetine.

### Baseline Demographic and Clinical Characteristics

Mean age was 59 years old, with a predominance of women and 2/3rds of the patients not working at onset of study (Table 2). Most patients (69.7%) were treated with more than one drug; paracetamol, opioids and non-steroidal anti-inflammatory drugs (NSAIDs) being the most frequent previous treatments with significant differences between groups (Table 2). Patients in PGB monotherapy group received a lower average number of previous treatments (p < 0.001). Both PGB groups presented with mean pain intensity significantly higher than the non-PGB group during the last week (VAS) and at the visit (PPI) as well; p < 0.001, indicating all of these findings a moderately worst baseline situation of subjects in groups receiving PGB.

### Drug Treatment during the Study

Most patients of non-PGB group (67%) received two or more drugs [mean (SD): 2.2 (1.2), p = 0.145 vs. pre-study number of drugs (table 3)], paracetamol being the most frequent (44% of subjects, mean dose: 2,144 ± 1,010 mg/day), followed by gabapentin (33%; 1,288 ± 543 mg/day), tramadol (29%; 214 ± 130 mg/day), ibuprofen (19%; 1,438 ± 517 mg/day), metamizol, an NSAID (17%; 1,679 ± 606 mg/day), amitriptyline (10%; 37 + 35 mg/day), diclofenac (7%; 145 + 69 mg/day), codeine (5%; 10 + 8 mg/day) or ketorolac (5%; 18 + 17 mg/day). Drugs used by less than 3% of patients are not shown. The mean dose of the group receiving PGB monotherapy was 208 ± 123 mg/day. The most frequently used drugs in the PGB add-on group (mean dose: 200 ± 113 mg/day) were paracetamol (40%; 1,866 ± 999 mg/day), tramadol (20%; 200 ± 111 mg/day), metamizol (19%; 1,428 ± 641 mg/day), ibuprofen (15%; 1,148 ± 502 mg/day), diclofenac (10%; 111+45 mg/day), amitriptyline (6%; 46+29 mg/day), gabapentin (4%; 1,023+630 mg/day), ketorolac (3%; 14+14 mg/day) and codeine (3%; 93+86 mg/day). In this group, the mean number of drugs was 2.7 (1.0), p = 0.159 (table 3).

**Table 3:** Use of drugs, non-pharmacological treatments, and complementary tests during the study by treatment group

Resource	Non-PGB **(N = 157)**^§^		PGB monotherapy (N = 577)^§^		PGB add-on (N = 575)^§^		p between groups	
	Baseline	Final	Baseline	Final	Baseline	Final	Baseline	**Final**^**$**^
**Drug treatment**								
Mean number (SD)	2.4 (1.3)	2.2 (1.2)	2.0 (1.1)	1.0 (0.0)*	2.6 (1.4)	2.7 (1.0)	<0.001	<0.001

**Non-pharmacological treatments**; N (%)								
Physiotherapy	45 (30.8)	23 (15.8)*	168 (34.1)	73 (14.8)*	194 (38.2)	116 (22.8)*	0.180	0.011
TENS	16 (11.4)	12 (8.6)	45 (9.6)	24 (5.1)*	52 (10.7)	32 (6.6)^┼^	0.766	0.375
Infiltrations	21 (14.9)	11 (7.8)^╪^	51 (10.8)	20 (4.2)*	72 (14.8)	21 (4.3)*	0.154	0.195
Electrotherapy	18 (12.9)	10 (7.1)^╪^	30 (6.5)	12 (2.6)*	48 (10.0)	17 (3.6)*	0.033	0.190
Blockade	3 (2.2)	1 (0.7)	8 (1.7)	4 (0.9)^╪^	5 (1.1)	2 (0.4)	0.552	0.858
Iontophoresis	4 (2.9)	0 (0.0)^╪^	1 (0.2)	0 (0.0)	7 (1.5)	1 (0.2)^╪^	0.019	0.530
Spinal stimulator	1 (0.7)	0 (0.0)	2 (0.4)	1 (0.2)	4 (0.8)	2 (0.4)	0.727	0.700
Pumps	1 (0.8)	0 (0.0)	0 (0.0)	0 (0.0)	3 (0.6)	0 (0.0)	0.214	1.000
Hydrotherapy	3 (1.8)	0 (0.0)	10 (1.7)	9 (1.5)	15 (2.5)	7 (1.2)	0.552	0.199
Short wave	3 (1.8)	1 (0.6)	7 (1.2)	1 (0.2)^╪^	22 (3.7)	12 (2.0)^╪^	0.013	0.053
Magnetotherapy	1 (0.6)	3 (1.8)	3 (0.5)	2 (0.3)	6 (1.0)	2 (0.3)	0.568	0.034
Acupuncture	1 (0.6)	0 (0.0)	4 (0.7)	0 (0.0)	3 (0.5)	0 (0.0)	0.938	1.000

**Complementary tests**; N (%)								
CT	25 (15.0)	17 (10.2)	155 (25.9)	39 (6.5)*	193 (32.8)	69 (11.7)*	<0.001	0.013
Resonance	60 (35.9)	32 (19.2)*	193 (32.3)	62 (10.4)*	224 (38.0)	88 (14.9)*	0.114	0.012
Electromyogram	40 (24.0)	22 (13.2)^╪^	164 (27.4)	51 (8.5)*	215 (36.5)	68 (11.5)*	0.001	0.109
ECHO Doppler	12 (7.2)	8 (4.8)	50 (8.4)	14 (2.3)*	59 (10.0)	20 (3.4)	0.424	0.194
Thermogram	6 (3.6)	1 (0.6)	4 (0.7)	3 (0.5)	13 (2.2)	10 (1.7)	0.016	0.151
X-rays	111 (66.5)	51 (30.5)*	363 (60.7)	123 (20.6)*	410 (69.6)	145 (24.6)*	0.005	0.058
General analysis	124 (74.3)	73 (43.7)*	427 (71.4)	198 (33.1)*	450 (76.4)	233 (39.6)*	0.146	0.032
Gammagram	12 (7.2)	5 (3.0)	41 (6.9)	11 (1.8)*	51 (8.7)	24 (4.1)*	0.491	0.129

SD: standard deviation; TENS: Transcutaneous electric neurostimulation; CT: Computerized tomography; * p < 0.0001; ^┼ ^p < 0.001; ^╪ ^^p^<0.05 vs. intragroup baseline value. ^§^Total number of analyzed patients; some patients did not report all data. ^**$**^Between groups changes comparison adjusted by baseline values and number of previous drugs.

### Pain Reduction

**Figure 2:**
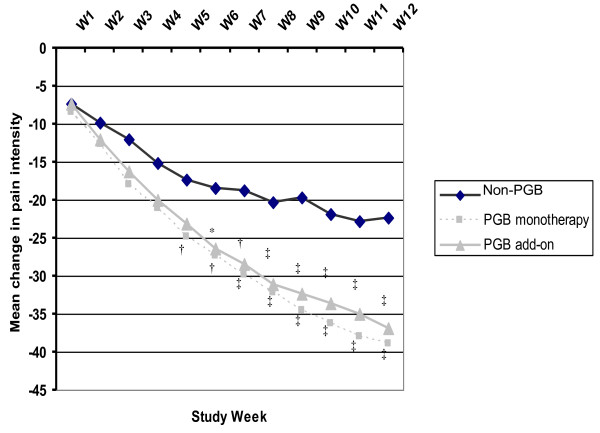
**Weekly mean change in pain intensity according to the SF-MPQ VAS**. VAS: Visual analogue scale; PGB: pregabalin; W1-W12: weeks 1 to 12; SF-MPQ: McGill Pain Questionnaire Short-Form. *p < 0.05, †p < 0.01, ‡p < 0.001 vs. Non-PGB group.

After adjusting for baseline scores, significant reductions in pain symptoms scores and intensity were observed in the three groups starting the first week of treatment (Figure 2), being this reduction significantly greater in both PGB groups compared to the non-PGB group, with mean changes of 54% and 51% for PGB monotherapy and add-on group, respectively, compared to 34% in the group not receiving PGB (p < 0.001). These significant differences were observed between the two PGB-treated groups and the non-PGB group from 5^th^/6^th ^weeks of follow up, which continued to decrease smoothly across the study (Figure 2). At the end of the study, 57.9% and 52.1% of patients who received PGB monotherapy and PGB add-on, respectively, showed a 50% reduction of baseline pain intensity, compared to 30.2% in the non-PGB group (p < 0.001), resulting in a higher cumulative number of days with no or mild pain in the PGB groups compared to the group not receiving PGB; 35.0 (29.8), 29.8 (28.9) and 25.5 (29.4), respectively (p < 0.001).

### Direct healthcare and indirect resources utilization and associated costs

Table 3 shows the average number of drugs, non-pharmacological therapies and complementary tests used. All treatment groups demonstrated a significant reduction in physiotherapy, infiltrations, and electrotherapy use relative to study initiation, and indicated significant reductions of the complementary tests prescribed in the last 12 weeks, except for echo Doppler and thermograms. Mean medical visits, overall and by type of visit, as well as rates of hospitalization and work productivity losses expressed in LWDE, are shown in table 4. After correcting for baseline differences, the PGB-treated groups showed significantly greater reductions in visits and LWDE (Table 4). On average, the number of overall medical visits in the PGB-treated groups was reduced by 4.4 and 4.3 in the 12-weeks period, compared to a reduction of 2.4 visits in the group not treated with PGB (p = 0.001). LWDE reductions were significantly higher in the PGB groups than in the non-PGB, by approximately an adjusted average of 22 and 23 LWDE vs. 14 in subjects not receiving PGB (p = 0.005, table 4). This was mainly a consequence of higher significant reductions in means number of days working with pain, rather than a substantial increase of labour productivity or reduction of days of absenteeism which, nevertheless, showed a trend toward statistical significance in favour of groups treated with PGB (table 4).

**Table 4:** Baseline values and change on use of medical visits, hospitalizations, and decreased work productivity after 12 weeks by group.

Resource	Non-PGB **(N = 157)**^**§**^		PGB monotherapy **(N = 577)**^**§**^		PGB add-on **(N = 575)**^**§**^		p between groups	
**Number of medical visits**	**Baseline**	**Change**	**Baseline**	**Change**	**Baseline**	**Change**	**Baseline**	**Change**^**$**^
Total	8.8 (7.0)	-2.4 (6.3)	9.6 (8.1)	-4.3 (6.4)	10.3 (7.1)	-4.3 (6.3)	0.050	0.001
Primary care	5.9 (4.1)	-1.2 (4.5)	6.7 (4.9)	-2.3 (4.6)	7.1 (4.8)	-2.2 (4.3)	0.011	0.064
Pain unit	0.5 (0.9)	0.0 (0.8)	0.7 (1.6)	-0.5 (1.4)	0.6 (1.2)	-0.3 (1.1)	0.164	0.003
Specialist	1.4 (1.7)	-0.5 (1.5)	1.2 (1.5)	-0.8 (1.4)	1.5 (1.7)	-0.8 (1.7)	0.033	0.007
Emergency room	1.6 (2.8)	-0.7 (1.9)	1.5 (3.1)	-1.0 (2.3)	1.6 (2.4)	-1.0 (2.1)	0.847	0.012

**Hospitalized **(N, %)	11 (7.7)	4 (2.8)	9 (1.8)	1 (0.2) ^╪^	34 (6.4)	14 (2.6) ^┼^	0.001	0.074

**Productivity**	**Baseline**	**Change**	**Baseline**	**Change**	**Baseline**	**Change**	**Baseline**	**Change**
Days of absenteeism due to pain	15.7 (21.7)	-5.4 (18.7) ^┼^	19.1 (23.4)	-10.4 (18.8)*	24.4 (26.9)	-12.9 (20.4)*	0.001	0.059
Days working with pain	35.0 (29.0)	-11.8 (26.6)*	40.3 (30.2)	-23.7 (28.6)*	43.2 (29.9)	-22.6 (28.6)*	0.012	0.001
Labour productivity^£^	48.1 (21.3)	+16.8 (79.7) ^╪^	46.9 (21.1)	+21.6 (24.0)*	43.4 (21.6)	+18.7 (23.2)*	0.007	0.064
LWDE	31.4 (25.9)	-14.0 (22.8)*	35.9 (26.7)	-22.1 (23.2)*	41.6 (28.3)	-23.1 (24.3)*	<0.001	0.005

Values expressed as means (standard deviation); LWDE: Lost -workdays equivalents; * p < 0.0001; ^┼ ^p < 0.001; ^╪ ^^p^<0.05 vs. intragroup baseline values. ^§^Total number of analyzed patients; some patients did not report all data; ^**$**^Between groups changes comparison adjusted by baseline values and number of previous drugs. ^£^In days working with pain.

The reduction of the use of direct healthcare and indirect resources resulted in a significant reduction of the overall costs despite the significant increases of drug costs in all of the three groups (Table 5). These increases, however, were compensated with significant adjusted reductions in direct, indirect and overall costs, being significantly higher in PGB groups (Table 5). The reduction in direct healthcare costs was meaningful in all of the groups analyzed, but significantly higher in non-PGB and PGB add-on groups when compared to the PGB monotherapy group (p = 0.019, Table 5).

**Table 5:** Overall and by components 12-weeks costs expressed in year 2006 Euros.

Costs (€)	Non-PGB (N = 157)		PGB monotherapy (N = 577)		PGB add-on (N = 575)		p between groups	
	**Baseline**	**Change**	**Baseline**	**Change**	**Baseline**	**Change**	**Baseline**	**Change**^**§**^

Pharmacological treatment	66.8 (93.1)	+34.6 (80.3)*	82.3 (106.9)	+160.7 (123.9)*	96.8 (120.2)	+154.5 (133.0)*	0.004	<0.001
Non-pharmacological treatment	258.8 (1211.6)	-191.1 (1,185.5) ^╪^	168.5 (525.9)	-121.9 (494.2)*	290.9 (983.1)	-223.6 (948.8)*	0.043	0.220
Medical visits and hospitalizations	430.3 (702.8)	-177.0 (695.3) ^┼^	351.3 (572.6)	-203 (485.4)*	497.5 (1,044.6)	-250.2 (879.8)*	0.010	0.058
Complementary tests	228.2 (257.0)	-104.3 (282.4)*	231.5 (247.2)	-158.1 (250.4)*	282.1 (254.9)	-173.4 (261.1)*	0.001	0.001

Total direct costs	984.1 (1,684.3)	-437.8 (1,582.6) ^┼^	833.6 (934.4)	-322.4 (812.9)*	1,167.3 (1,666.4)	-492.7 (1,491.4)*	0.001	0.019
Indirect costs (LWDE)	1,412.5 (1,339.7)	-607.5 (1,141.1)*	1,636.1 (1,382.9)	-990.5 (1,187.9)*	1,942.2 (1,485.4)	-1,072.8 (1,211.7)*	<0.001	0.005

**Total costs**	2,396.6 (2,308.0)	-1,045.3 (1,989.6)	2,469.7 (1,856.8)	-1,312.9 (1,543.0)*	3,109.5 (2,495.8)	-1,565.5 (2,004.1)*	<0.001	0.030

Values expressed as means (Standard deviation). *p < 0.0001; ^┼^p < 0.001; ^╪ ^p < 0.05 vs. intragroup baseline values; LWDE = Lost-workdays equivalents. ^**§**^Between groups changes comparison adjusted by baseline values and number of previous drugs.

## Discussion

To date, data have been published about economic evaluation of oral therapies for peripheral NeP disorders, including modelling the cost-effectiveness of gabapentin and PGB [33-37]. However, data presented here are the first to evaluate the effect of PGB on cost and consequences of the treatment of NeP of peripheral origin in routine clinical practice conditions ("the Real World") and, thus, complementing the findings from previous clinical trial data. PGB, monotherapy and add-on therapy, administered at doses within the therapeutically recommended range, produced a marked reduction of pain (over 50%). Percentage of patient responders were very similar to those reported in published clinical trials of PGB in patients with diabetic neuropathy [38-40], and post-herpetic neuralgia [21-23,41]. Variability in mean PGB doses observed in both PGB groups reinforces the absence, in clinical practice, of a single drug, or a single effective dose suitable for all NeP patients. This point is supported by similar variability observed in mean doses of medications used in the group not receiving PGB [42]. The fact that PGB dose recorded in this study was at the bottom of its therapeutic range could explain the different declining profile of pain observed in this observational trial when compared with the findings observed in randomized clinical trials in which a more rapid decline in the first weeks of treatment, followed by a plateau of effect, was observed [21-23,38-42].

The reduction observed in baseline pain score translated into a substantial decrease of the use of both healthcare and indirect resources, resulting in a subsequent reduction in overall costs. The component of cost related to drug costs increased significantly in all groups, particularly with PGB. However, these increases were offset by significant reductions in healthcare and indirect costs, yielding a significant reduction in overall costs. Decrease in direct costs was more marked in the group of subjects not receiving PGB and in the PGB add-on group than in the PGB monotherapy group. However, indirect costs, derived from the lost-workdays equivalents across the 12 week period, were also reduced at final visit to a greater extent than direct healthcare costs: the magnitude of the reduction being significantly greater in the PGB-treated groups. As a result, mean reductions in total costs were higher in both PGB groups than in patients not receiving PGB. These reductions ranged between 62% and 76% over the non-PGB group, and should be considered of a meaningful size effect.

Interestingly, the mean utilization of all-type medical visits was significantly reduced in all study groups, although this reduction was again more marked in the PGB-treated groups compared with the non-PGB group. Over 50% of the reduction in total visits was due to decrease of the number of visits to primary care doctors. This might be of relevance, taking into account the increasingly longer waiting lists in most primary care clinics. Furthermore, use of complementary tests was reduced by over 50% for most items listed. Except for the PGB monotherapy group that by definition received one drug only, the mean number of prescribed drugs remained hardly unchanged in the two other groups throughout the study.

The magnitudes of baseline costs observed in this trial were similar to those observed in other studies conducted in our context [11,34] or in Canada [35]. These results were within the range of values observed in our observational study, supporting the validity of "real world" health costs obtained in the present study. While some economic evaluations of clinical trial data in NeP have been published comparing diverse medical interventions in subjects with peripheral NeP, we could not find any studies investigating real world practice conditions in NeP, including those studying PGB [43].

Our study presents, however, some limitations that should be borne in mind. Among them, the observational design of the study implies potential confounding factors. One of these factors is confounding by indication, inherent to observational studies involving drugs [44]. This would explain, for instance, the significant differences observed among the three groups selected for the analysis of their baseline clinical characteristics, use of complementary tests, mean number of medical visits, particularly to primary care and specialist visits, as well as LWDE. However, these differences would be expected to bias the results against PGB since, as these patients had higher levels of baseline pain severity, significantly more LWDEs, more prescribed complementary tests, or more medical visits, yielding to higher quarterly costs in cohort receiving PGB as an add-on therapy. Then, certain risk for residual confounding could not be ruled out. In the PGB monotherapy group, this might be explained by a lower use of drugs in previous therapeutic schedules, or to a lower use of opioids before the study, compared to higher levels of previous exposure to NSAIDs and paracetamol in the other two groups. In general, subjects receiving PGB in monotherapy were exposed to lower percentages of analgesics, except tricyclic drugs, than the other two study groups. On the opposite, patients in the PGB add-on group received lower levels of exposure to antiepileptic drugs. These could be the only explanation for mentioned baseline differences, as groups were similar in distribution of type of neuropathic pain, elapsed time from diagnosis and the rest of demographic characteristics collected in the trial. It appeared that subjects receiving PGB as monotherapy could be less resistant patients. Other possible limitation is the unbalanced sample size of non-PGB group in comparison with the other two groups reflecting a possible selection preference of participants for pregabalin. However, and as mentioned, bias could go against pregabalin given the worst profile of subjects in some variables and the statistical analysis performed dealt with this unbalanced sample size of groups. Another limitation is that the study was not able to capture out-of-pocket cost, thus, a full societal perspective economic evaluation could not be performed. However, the economic impact of this limitation on overall costs is really limited due to the nature of NeP as most direct healthcare resources consumption are financed by Social Security and indirect costs represent the largest portion of total cost in these conditions. Also, it is worthy to comment on the diagnosis of NeP in the study. While patients were identified using ICD-10 classification criteria for peripheral NeP in conjunction with a diagnostic tool administered to assist general practitioners in categorizing the neuropathic component of pain, we cannot exclude the possibility of misdiagnosis to some extent. On the other hand, to calculate LWDE, the study recorded patient's self-perceived productivity, which could incorporate some degree of bias or uncertainty. The etiological diagnosis of types of NeP included here may have different long-term evolutions both in term of outcomes follow-up and health resources utilization and corresponding costs. Due this, any findings observed in this research should be limited to the trial duration of this study.

Patients in non-PGB and PGB add-on therapy groups were receiving analgesics without indication for neuropathic pain, such as paracetamol, metamizol or NSAIDs to some extent. The use of these non-appropriate therapies could explain partially the lower effectiveness and higher resources utilization observed in the results of non-PGB group; even they were treated with appropriate therapies in at least three out of four cases (near 77% of subjects in this group were treated with gabapentin, tramadol, codeine or amitriptyline, perhaps at doses in the lower end of its therapeutic range). On the opposite, all subjects included in PGB groups received an appropriate analgesic (an analgesic indicated for the treatment of neuropathic pain) by definition.

Overall, despite these limitations and the fact that a residual confounding can not be completely ruled out, the results of this analysis complement the findings observed with PGB in clinical trials, then consolidating PGB as an effective therapy for the treatment of peripheral NeP due to diabetic neuropathy, postherpetic neuralgia or trigeminal neuralgia in real world conditions of care. This effectiveness resulted in a reduction of the use of direct healthcare and indirect resources in routine medical practice, leading to lower costs both for the National Health System and society. Other learning from this trial was the observation of inadequate use, type and/or doses, of analgesics for neuropathic pain conditions in the real world in an important proportion of subjects.

## Conclusion

To conclude, our analysis suggests that treatment with pregabalin both, in monotherapy or in combination with other medications produces a substantial reduction in pain in subjects with peripheral NeP, resulting in a substantial decrease in overall costs during the 12-week period of the study. The drug cost was largely counterbalanced by a greater cost reduction in other components of the healthcare system, and by a significant reduction in LWDE. However, randomized pragmatic clinical trials should be conducted to confirm these findings.

## Competing interests

Ana Navarro, M^a ^Teresa Saldaña and Concepción Pérez have received honoraries from Pfizer Spain. Sandra Torrades is an employee of a CRO which works for Pfizer Spain.

Javier Rejas is an employee of Pfizer Spain.

## Authors' contributions

This was a collaborative project, and the authors worked closely together. CP, MTS, AN and JR participated in the design of the original study and in the interpretation of data and drafting of the manuscript. ST carried out the analysis and participated in the interpretation of data and in the preparation of the manuscript. All authors were responsible for the literature review and extraction of references.

## Pre-publication history

The pre-publication history for this paper can be accessed here:

http://www.biomedcentral.com/1471-2377/11/7/prepub

## Supplementary Material

Additional file 1 **LIDO study Case Report Form**. This file contents the original case report form used in the LIDO study (in Spanish). Click here for file
