# Supplementary material for: A Cost-Consequences analysis of the effect of Pregabalin in the treatment of peripheral Neuropathic Pain in routine medical practice in Primary Care settings
Source: BMC Neurol. 2011 Jan 20;11:7. doi: 10.1186/1471-2377-11-7 (PMC3037328; doi:10.1186/1471-2377-11-7)
Supplement: Additional file 1 — LIDO study Case Report Form. This file contents the original case report form used in the LIDO study (in Spanish). [file 1471-2377-11-7-S1.PDF]

A0081109 - Estudio LIDO

Iniciales: ☐☐☐

Nº PACIENTE ☐

Visita Basal

FECHA DE VISITA ☐☐ - ☐☐ - 2 0 ☐☐

DD MM AAAA

Form 1 - CRITERIOS DE SELECCIÓN

CUESTIONARIO DN4

Responda a las cuatro preguntas siguientes marcando Sí o No en la casilla correspondiente

ENTREVISTA AL PACIENTE

Pregunta 1: ¿Tiene su dolor alguna de estas características?

Quemazón	1 ☐ Sí	2 ☐ No
Sensación de frío doloroso	1 ☐ Sí	2 ☐ No
Descargas eléctricas	1 ☐ Sí	2 ☐ No

Pregunta 2: ¿Tiene en la zona donde le duele alguno de estos síntomas?

Hormigueo	1 ☐ Sí	2 ☐ No
Pinchazos	1 ☐ Sí	2 ☐ No
Entumecimiento	1 ☐ Sí	2 ☐ No
Escozor	1 ☐ Sí	2 ☐ No

EXPLORACIÓN DEL PACIENTE

Pregunta 3: ¿Se evidencia en la exploración alguno de estos signos en la zona dolorosa?

Hipoestesia al tacto	1 ☐ Sí	2 ☐ No
Hipoestesia al pinchazo	1 ☐ Sí	2 ☐ No

Pregunta 4: ¿El dolor se provoca o intensifica por?

El roce	1 ☐ Sí	2 ☐ No

CRITERIOS DE INCLUSIÓN

• Pacientes de ambos sexos, mayores de 18 años.	1 ☐ Sí	2 ☐ No
• Pacientes con dolor crónico de más de 6 meses de duración	1 ☐ Sí	2 ☐ No
• Pacientes con dolor neuropático asociado a una de las siguientes patologías:<br>• Neuropatía diabética:<br>- Paciente con diabetes mellitus tipo 1 y 2 en tratamiento con insulina diaria o un hipoglucemiante oral<br>- Presencia de dolor crónico en pies y/o manos<br>• Neuralgia postherpética:<br>- Presencia de dolor como secuela de una infección por Herpes Zoster<br>• Neuralgia del Trigémino:<br>- Dolor en forma de crisis paroxísticas en la zona del quinto par craneal	1 ☐ Sí	2 ☐ No
• Pacientes con 4 o más respuestas afirmativas en el cuestionario DN4	1 ☐ Sí	2 ☐ No
• Pacientes con suficiente nivel cultural y educativo para cumplimentar cuestionarios de salud	1 ☐ Sí	2 ☐ No
• Pacientes que autorizan oralmente su inclusión en el estudio	1 ☐ Sí	2 ☐ No

SI HA CONTESTADO "NO" A ALGUNO DE LOS CRITERIOS NO DEBE INCLUIRSE AL PACIENTE EN EL ESTUDIO.

Area de Biometría
Instituto Euroclin/Pfizer

A0081109 - Estudio LIDO

Iniciales:

Nº PACIENTE

Visita Basal

Form 2 - DATOS DEL PACIENTE

DATOS SOCIODEMOGRÁFICOS

Fecha de Nacimiento: __ / __ / ____

Peso: ___,_ kg.

Sexo: 1 ☐ Hombre 2 ☐ Mujer

Talla: ___ cm.

Nivel de estudios:
1 ☐ Sin estudios
2 ☐ Enseñanza primaria
3 ☐ Enseñanza secundaria
4 ☐ Estudios medios
5 ☐ Estudios superiores

Estado civil:
1 ☐ Soltero/a
2 ☐ Casado/a o en pareja
3 ☐ Separado/a o divorciado/a
4 ☐ Viudo/a

Situación laboral:

Si el sujeto es "ama de casa" y no ejerce ningún trabajo remunerado, marcar la casilla "Otra" y especificarlo.

1 ☐ Activo
2 ☐ Parado
3 ☐ Baja por incapacidad laboral
4 ☐ Jubilado
5 ☐ No ejerce
6 ☐ Otra (especificar) ____________________________
9 ☐ NS/NC

DIAGNÓSTICO DEL DOLOR NEUROPÁTICO

Fecha del diagnóstico: __ / __ / ____

PRODUCTIVIDAD

En los últimas 3 meses, ¿cuántos días ha dejado de trabajar o de realizar sus actividades cotidianas debido a su dolor?

Nº de días: __

En los últimos 3 meses, ¿cuántos días ha tenido que acudir a su trabajo o ha realizado sus actividades cotidianas con síntomas u otros problemas relacionados con su dolor?

Nº de días: __

En relación a la pregunta anterior, ¿cómo describiría su rendimiento promedio en el trabajo o en sus actividades cotidianas en esos días?

0%	10%	20%	30%	40%	50%	60%	70%	80%	90%	100%
☐	☐	☐	☐	☐	☐	☐	☐	☐	☐	☐

Rendimiento nulo — Pleno rendimiento

Pfizer **A0081109 - Estudio LIDO**

Iniciales: ☐☐☐ Nº PACIENTE ☐

Visita Basal

Form 3 - UTILIZACIÓN DE RECURSOS SANITARIOS

TRATAMIENTO FARMACOLÓGICO (a causa de su dolor) EN LOS ÚLTIMOS 3 MESES

Nombre del Fármaco (marca comercial)	Dosis	Fecha de Inicio (dd/mm/aa)	Fecha Fin (dd/mm/aa)
		/ /	/ /
		/ /	/ /
		/ /	/ /
		/ /	/ /
		/ /	/ /

TRATAMIENTO NO FARMACOLÓGICO (a causa de su dolor) EN EL ÚLTIMO AÑO

Fisioterapia .... 2 ☐ No 1 ☐ Sí ➩ Nº sesiones:

TENS .... 2 ☐ No 1 ☐ Sí ➩ Nº sesiones:

Infiltraciones (por ej. articulación) .... 2 ☐ No 1 ☐ Sí ➩ Nº sesiones:

Electroterapia .... 2 ☐ No 1 ☐ Sí ➩ Nº sesiones:

Bloqueo (por ej. epidural) .... 2 ☐ No 1 ☐ Sí

Iontoforesis .... 2 ☐ No 1 ☐ Sí

Estimulador medular .... 2 ☐ No 1 ☐ Sí

Bombas .... 2 ☐ No 1 ☐ Sí

Otras (por ej. magnetografía, hidroterapia, microondas):

2 ☐ No 1 ☐ Sí ➩ Nº sesiones:

VISITAS MÉDICAS (a causa de su dolor) EN LOS ÚLTIMOS 3 MESES

A Atención Primaria Nº de visitas: Al especialista Nº de visitas:

A la Unidad de Dolor Nº de visitas: A Urgencias Nº de visitas:

HOSPITALIZACIONES (a causa de su dolor) EN LOS ÚLTIMOS 3 MESES

Hospitalizaciones 2 ☐ No 1 ☐ Sí ➩ Nº total de días hospitalizados:

PRUEBAS COMPLEMENTARIAS (a causa de su dolor) EN EL ÚLTIMO AÑO

	REALIZADA	SOLICITADA		REALIZADA	SOLICITADA
TAC	1 ☐	1 ☐	Termografía	1 ☐	1 ☐
Resonancia	1 ☐	1 ☐	Radiografía	1 ☐	1 ☐
Electromiograma	1 ☐	1 ☐	Analítica general	1 ☐	1 ☐
ECO dopler	1 ☐	1 ☐	Gammagrafía ósea	1 ☐	1 ☐

Otra (esp.): 1 ☐ 1 ☐

A0081109 - Estudio LIDO

Iniciales: | Nº PACIENTE | Página 4

Visita Basal

Form 4 - CUESTIONARIO McGILL

CUESTIONARIO ABREVIADO McGill

A. Describa su dolor durante la última semana (marque una casilla con una "X" en cada línea).

1. Como pulsaciones:	1 ☐ No	2 ☐ Leve	3 ☐ Moderado	4 ☐ Severo	9. Pesado:	1 ☐ No	2 ☐ Leve	3 ☐ Moderado	4 ☐ Severo
2. Como una sacudida:	1 ☐ No	2 ☐ Leve	3 ☐ Moderado	4 ☐ Severo	10. Escozor:	1 ☐ No	2 ☐ Leve	3 ☐ Moderado	4 ☐ Severo
3. Como un latigazo:	1 ☐ No	2 ☐ Leve	3 ☐ Moderado	4 ☐ Severo	11. Como un desgarro:	1 ☐ No	2 ☐ Leve	3 ☐ Moderado	4 ☐ Severo
4. Pinchazo:	1 ☐ No	2 ☐ Leve	3 ☐ Moderado	4 ☐ Severo	12. Que consume:	1 ☐ No	2 ☐ Leve	3 ☐ Moderado	4 ☐ Severo
5. Calambre:	1 ☐ No	2 ☐ Leve	3 ☐ Moderado	4 ☐ Severo	13. Que marea:	1 ☐ No	2 ☐ Leve	3 ☐ Moderado	4 ☐ Severo
6. Retortijón:	1 ☐ No	2 ☐ Leve	3 ☐ Moderado	4 ☐ Severo	14. Temible:	1 ☐ No	2 ☐ Leve	3 ☐ Moderado	4 ☐ Severo
7. Ardiente o quemante:	1 ☐ No	2 ☐ Leve	3 ☐ Moderado	4 ☐ Severo	15. Que atormenta:	1 ☐ No	2 ☐ Leve	3 ☐ Moderado	4 ☐ Severo
8. Entumecimiento:	1 ☐ No	2 ☐ Leve	3 ☐ Moderado	4 ☐ Severo					

B

Valore su DOLOR durante la última semana

La línea presentada a continuación representa el dolor en orden creciente de intensidad, desde "no dolor" (0) hasta "dolor extremo" (10). Marque con una línea (/) la posición que mejor describa su dolor durante la última semana.

____

0 1 2 3 4 5 6 7 8 9 10

C

Intensidad del DOLOR en este momento

1 ☐ Nada de dolor	4 ☐ Intenso
2 ☐ Leve	5 ☐ Horrible
3 ☐ Molesto	6 ☐ Insufrible

CUESTIONARIOS PARA EL PACIENTE

Recuerde que el paciente debe rellenar en ESTA VISITA los cuestionarios de las páginas 5-8. Por favor, revise que los ha completado correctamente

REGISTRO SEMANAL

No olvide entregar al paciente el REGISTRO SEMANAL DEL DOLOR Y DEL ESTADO DE SALUD y recuérdele que debe traerlo en la próxima visita del estudio

A0081109 - Estudio LIDO

Iniciales:

Nº PACIENTE

Visita Basal

Form 5 - CUESTIONARIOS PARA EL PACIENTE (1 de 4)

CUESTIONARIO DE INCAPACIDAD DE SHEEHAN

Señale con una cruz en el número que describa mejor su situación actual.

A causa de sus síntomas su trabajo se ha visto perjudicado

No, en absoluto — Levemente — Moderadamente — Marcadamente — Extremadamente

☐ 0 ☐ 1 ☐ 2 ☐ 3 ☐ 4 ☐ 5 ☐ 6 ☐ 7 ☐ 8 ☐ 9 ☐ 10

A causa de sus síntomas su vida social y sus actividades de tiempo libre se han visto perjudicadas

No, en absoluto — Levemente — Moderadamente — Marcadamente — Extremadamente

☐ 0 ☐ 1 ☐ 2 ☐ 3 ☐ 4 ☐ 5 ☐ 6 ☐ 7 ☐ 8 ☐ 9 ☐ 10

A causa de sus síntomas su vida familiar y sus responsabilidades domésticas se han visto perjudicadas

No, en absoluto — Levemente — Moderadamente — Marcadamente — Extremadamente

☐ 0 ☐ 1 ☐ 2 ☐ 3 ☐ 4 ☐ 5 ☐ 6 ☐ 7 ☐ 8 ☐ 9 ☐ 10

ESTRÉS PERCIBIDO

Desde su última visita, ¿cuánto le han dificultado la vida los eventos estresantes y los problemas personales como los del trabajo, la casa, de salud o económicos?

No, en absoluto — Levemente — Moderadamente — Marcadamente — Extremadamente

☐ 0 ☐ 1 ☐ 2 ☐ 3 ☐ 4 ☐ 5 ☐ 6 ☐ 7 ☐ 8 ☐ 9 ☐ 10

APOYO SOCIAL PERCIBIDO

Durante la última semana, ¿qué porcentaje de apoyo ha recibido de amigos, familiares, compañeros de trabajo, etc., respecto al apoyo que ha necesitado?

Ningún apoyo en absoluto — Un poco — Moderado — Considerable — El apoyo ideal

☐ 0% ☐ 10 ☐ 20 ☐ 30 ☐ 40 ☐ 50 ☐ 60 ☐ 70 ☐ 80 ☐ 90 ☐ 100%

Pfizer **A0081109 - Estudio LIDO**

Iniciales: ☐☐☐ Nº PACIENTE ☐

Visita Basal

Form 5 - CUESTIONARIOS PARA EL PACIENTE (2 de 4)

ESCALA HOSPITALARIA DE DEPRESIÓN Y ANSIEDAD (HAD)

Los médicos son conscientes de que las emociones juegan un papel importante en la mayoría de las enfermedades. Si su médico se informa de estos estado de ánimo podrá ayudarle mejor.

Este cuestionario se ha diseñado para ayudar a que su médico sepa cómo se siente usted. No tome en cuenta los números impresos al margen del cuestionario. Lea cada sección y marque con una cruz la respuesta que más se ajuste a su estado de ánimo durante la última semana. No piense mucho las respuestas. Probablemente una reacción espontánea ante cada pregunta refleje mejor sus verdaderos sentimientos que una respuesta meditada durante mucho tiempo.

1. Me siento tenso/a o "nervioso/a":

3 ☐ La mayor parte del tiempo   2 ☐ Muchas veces   1 ☐ De vez en cuando, de repente   0 ☐ Nunca

2. Todavía disfruto con lo que antes me gustaba:

0 ☐ Como siempre   1 ☐ Un poco menos   2 ☐ Sólo un poco   3 ☐ Casi nada

3. Me viene una sensación de miedo, como si algo terrible me fuera a suceder:

3 ☐ Definitivamente y es muy fuerte   2 ☐ Sí, pero no es muy fuerte   1 ☐ Un poco, pero me preocupa   0 ☐ Nada

4. Puedo reírme y me hace gracia el aspecto divertido de las cosas:

0 ☐ Al igual que siempre lo hacía   1 ☐ Un poco menos ahora   2 ☐ Categóricamente creo que menos   3 ☐ Nunca

5. Tengo la mente llena de preocupaciones:

3 ☐ Gran parte del tiempo   2 ☐ Con bastante frecuencia   1 ☐ A veces, aunque no muy a menudo   0 ☐ Sólo en ocasiones

6. Me siento alegre:

3 ☐ Nunca   2 ☐ No muy a menudo   1 ☐ A veces   0 ☐ Casi siempre

7. Puedo descansar y relajarme:

0 ☐ Sí, no tengo ningún problema al respecto   1 ☐ Por lo general   2 ☐ No muy a menudo   3 ☐ Nunca

8. Me da la impresión que me demoro más que antes en hacer las cosas:

3 ☐ Prácticamente en todo momento   2 ☐ Muy a menudo   1 ☐ A veces   0 ☐ Nunca

9. Me viene una sensación de miedo, como un vacío en el estómago:

0 ☐ Nunca   1 ☐ En ciertas ocasiones   2 ☐ Con bastante frecuencia   3 ☐ Muy a menudo

10. He perdido interés en mi aspecto físico:

3 ☐ Categóricamente, sí   2 ☐ No me preocupa tanto como debiera   1 ☐ Quizá no me preocupe tanto como antes   0 ☐ Me preocupo al igual que siempre

11. Me siento inquieto/a, como si necesitara estar en constante movimento:

3 ☐ Mucho   2 ☐ Bastante   1 ☐ No mucho   0 ☐ Nada

12. Me siento optimista respecto a las cosas que están por venir:

0 ☐ Igual que siempre   1 ☐ Menos de lo que acostumbraba   2 ☐ Mucho menos de lo que acostumbraba   3 ☐ Casi nada

13. Me asaltan sentimientos repentinos de pánico:

3 ☐ Con muchísima frecuencia   2 ☐ Bastante a menudo   1 ☐ No muy a menudo   0 ☐ Nunca

14. Me divierto con un buen libro, la radio, o un programa de televisión:

0 ☐ A menudo   1 ☐ A veces   2 ☐ No muy a menudo   3 ☐ Rara vez

Compruebe ahora que ha contestado a todas las preguntas

Visita Basal

Form 5 - CUESTIONARIOS PARA EL PACIENTE (3 de 4)

CUESTIONARIO DE SALUD EuroQoL-5D (EQ-5D)

Marque con una cruz la respuesta de cada apartado que mejor describa su estado de salud *en el día de hoy*

Movilidad

1 ☐ No tengo problemas para caminar

2 ☐ Tengo algunos problemas para caminar

3 ☐ Tengo que estar en la cama

Cuidado personal

1 ☐ No tengo problemas con el cuidado personal

2 ☐ Tengo algunos problemas para lavarme o vestirme

3 ☐ Soy incapaz de lavarme o vestirme

Actividades cotidianas (p. ej., trabajar, estudiar, hacer las tareas domésticas, actividades familiares o durante el tiempo libre)

1 ☐ No tengo problemas para realizar mis actividades cotidianas

2 ☐ Tengo algunos problemas para realizar mis actividades cotidianas

3 ☐ Soy incapaz de realizar mis actividades cotidianas

Dolor / malestar

1 ☐ No tengo dolor ni malestar

2 ☐ Tengo moderado dolor o malestar

3 ☐ Tengo mucho dolor o malestar

Ansiedad / depresión

1 ☐ No estoy ansioso ni deprimido

2 ☐ Estoy moderadamente ansioso o deprimido

3 ☐ Estoy muy ansioso o deprimido

Comparado con mi estado general de salud durante los últimos 12 meses, mi estado de salud hoy es:
(POR FAVOR, PONGA UNA CRUZ EN EL CUADRO)

1 ☐ Mejor

2 ☐ Igual

3 ☐ Peor

Para ayudar a la gente a describir lo bueno y lo malo que es su estado de salud hemos dibujado una escala parecida a un termómetro en la cual se marca con un 100 el mejor estado de salud que se pueda imaginar y con un 0 el peor estado de salud que se pueda imaginar.

Nos gustaría que nos indicara en esta escala, en su opinión, lo bueno o malo que es su estado de salud en el día de *hoy*.

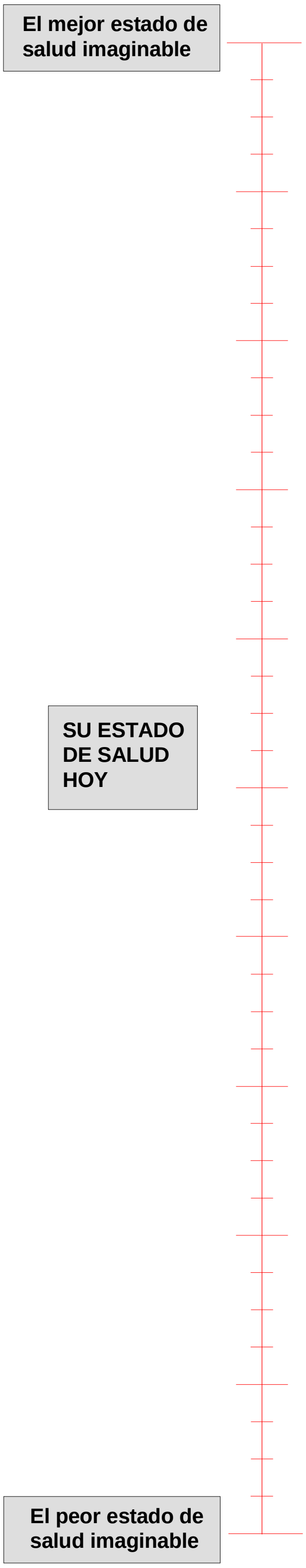

A0081109 - Estudio LIDO

Iniciales: ☐☐☐ Nº PACIENTE ☐

Visita Basal

Form 5 - CUESTIONARIOS PARA EL PACIENTE (4 de 4)

MÓDULO DE SUEÑO DEL M.O.S.

SU SUEÑO

	0-15 min.	16-30 min.	31-45 min.	46-60 min.	más de 60 min.
1. ¿Cuánto tiempo ha tardado habitualmente en dormirse durante las 4 últimas semanas? (marque un solo número)	☐ 1	☐ 2	☐ 3	☐ 4	☐ 5

2. De promedio, ¿cuántas horas ha dormido **cada noche** durante **las 4 últimas semanas**?

|__|__| Escriba el número de horas por noche

¿Con qué frecuencia durante las 4 últimas semanas?...

(Marque un solo número por cada pregunta)

	Siempre	Casi siempre	Muchas veces	Algunas veces	Sólo alguna vez	Nunca
3. ¿ha notado que su sueño no era tranquilo (moviéndose de forma inquieta sintiéndose tenso/a, hablando, etc, mientras dormía)?	☐ 1	☐ 2	☐ 3	☐ 4	☐ 5	☐ 6
4. ¿ha dormido lo suficiente como para sentirse descansado/a al despertarse por la mañana?	☐ 1	☐ 2	☐ 3	☐ 4	☐ 5	☐ 6
5. ¿se ha despertado con falta de aire o con dolor de cabeza?	☐ 1	☐ 2	☐ 3	☐ 4	☐ 5	☐ 6
6. ¿se ha sentido soñoliento/a o dormido/a durante el día?	☐ 1	☐ 2	☐ 3	☐ 4	☐ 5	☐ 6
7. ¿ha tenido problemas para quedarse dormido/a?	☐ 1	☐ 2	☐ 3	☐ 4	☐ 5	☐ 6
8. ¿se ha despertado mientras dormía y le ha costado volverse a dormir?	☐ 1	☐ 2	☐ 3	☐ 4	☐ 5	☐ 6
9. ¿le ha costado mantenerse despierto/a durante el día?	☐ 1	☐ 2	☐ 3	☐ 4	☐ 5	☐ 6
10. ¿ha roncado mientras dormía?	☐ 1	☐ 2	☐ 3	☐ 4	☐ 5	☐ 6
11. ¿se ha echado siestas (de 5 minutos o más) durante el día?	☐ 1	☐ 2	☐ 3	☐ 4	☐ 5	☐ 6
12. ¿ha dormido todo lo que necesitaba?	☐ 1	☐ 2	☐ 3	☐ 4	☐ 5	☐ 6

A0081109 - Estudio LIDO

Iniciales: ___ ___ ___ Nº PACIENTE ______ Página 9

Visita Final (Semana 12)

FECHA DE VISITA __ __ - __ __ - 2 0 __ __
DD MM AAAA

Form 6 - UTILIZACIÓN DE RECURSOS SANITARIOS

TRATAMIENTO FARMACOLÓGICO (a causa de su dolor) EN LOS ÚLTIMOS 3 MESES

Nombre del Fármaco (marca comercial)	Dosis	Fecha de Inicio (dd/mm/aa)	Fecha Fin (dd/mm/aa)
		/ /	/ /
		/ /	/ /
		/ /	/ /
		/ /	/ /
		/ /	/ /

TRATAMIENTO NO FARMACOLÓGICO (a causa de su dolor) EN LOS ÚLTIMOS 3 MESES

Fisioterapia .... 2 ☐ No 1 ☐ Sí ➪ Nº sesiones: ___

TENS .... 2 ☐ No 1 ☐ Sí ➪ Nº sesiones: ___

Infiltraciones (por ej. articulación) .... 2 ☐ No 1 ☐ Sí ➪ Nº sesiones: ___

Electroterapia .... 2 ☐ No 1 ☐ Sí ➪ Nº sesiones: ___

Bloqueo (por ej. epidural) .... 2 ☐ No 1 ☐ Sí

Iontoforesis .... 2 ☐ No 1 ☐ Sí

Estimulador medular .... 2 ☐ No 1 ☐ Sí

Bombas .... 2 ☐ No 1 ☐ Sí

Otras (por ej. magnetografía, hidroterapia, microondas):

___________ 2 ☐ No 1 ☐ Sí ➪ Nº sesiones: ___

VISITAS MÉDICAS (a causa de su dolor) EN LOS ÚLTIMOS 3 MESES

A Atención Primaria Nº de visitas: ___ Al especialista Nº de visitas: ___

A la Unidad de Dolor Nº de visitas: ___ A Urgencias Nº de visitas: ___

HOSPITALIZACIONES (a causa de su dolor) EN LOS ÚLTIMOS 3 MESES

Hospitalizaciones 2 ☐ No 1 ☐ Sí ➪ Nº total de días hospitalizados: ___

PRUEBAS COMPLEMENTARIAS (a causa de su dolor) EN LOS ÚLTIMOS 3 MESES

	REALIZADA	SOLICITADA		REALIZADA	SOLICITADA
TAC	1 ☐	1 ☐	Termografía	1 ☐	1 ☐
Resonancia	1 ☐	1 ☐	Radiografía	1 ☐	1 ☐
Electromiograma	1 ☐	1 ☐	Analítica general	1 ☐	1 ☐
ECO dopler	1 ☐	1 ☐	Gammagrafía ósea	1 ☐	1 ☐
Otra (esp.): ___				1 ☐	1 ☐

A0081109 - Estudio LIDO

Iniciales: ☐☐☐

Nº PACIENTE ☐

Visita Final (Semana 12)

Form 7 - CUESTIONARIO McGILL Y PRODUCTIVIDAD

CUESTIONARIO ABREVIADO McGill

A. Describa su dolor durante la última semana (marque una casilla con una "X" en cada línea).

1. Como pulsaciones:	1 ☐ No	2 ☐ Leve	3 ☐ Moderado	4 ☐ Severo
2. Como una sacudida:	1 ☐ No	2 ☐ Leve	3 ☐ Moderado	4 ☐ Severo
3. Como un latigazo:	1 ☐ No	2 ☐ Leve	3 ☐ Moderado	4 ☐ Severo
4. Pinchazo:	1 ☐ No	2 ☐ Leve	3 ☐ Moderado	4 ☐ Severo
5. Calambre:	1 ☐ No	2 ☐ Leve	3 ☐ Moderado	4 ☐ Severo
6. Retortijón:	1 ☐ No	2 ☐ Leve	3 ☐ Moderado	4 ☐ Severo
7. Ardiente o quemante:	1 ☐ No	2 ☐ Leve	3 ☐ Moderado	4 ☐ Severo
8. Entumecimiento:	1 ☐ No	2 ☐ Leve	3 ☐ Moderado	4 ☐ Severo
9. Pesado:	1 ☐ No	2 ☐ Leve	3 ☐ Moderado	4 ☐ Severo
10. Escozor:	1 ☐ No	2 ☐ Leve	3 ☐ Moderado	4 ☐ Severo
11. Como un desgarro:	1 ☐ No	2 ☐ Leve	3 ☐ Moderado	4 ☐ Severo
12. Que consume:	1 ☐ No	2 ☐ Leve	3 ☐ Moderado	4 ☐ Severo
13. Que marea:	1 ☐ No	2 ☐ Leve	3 ☐ Moderado	4 ☐ Severo
14. Temible:	1 ☐ No	2 ☐ Leve	3 ☐ Moderado	4 ☐ Severo
15. Que atormenta:	1 ☐ No	2 ☐ Leve	3 ☐ Moderado	4 ☐ Severo

B

Valore su DOLOR durante la última semana

La línea presentada a continuación representa el dolor en orden creciente de intensidad, desde "no dolor" (0) hasta "dolor extremo" (10). Marque con una línea (/) la posición que mejor describa su dolor durante la última semana.

0 1 2 3 4 5 6 7 8 9 10

C

Intensidad del DOLOR en este momento

1 ☐ Nada de dolor
2 ☐ Leve
3 ☐ Molesto
4 ☐ Intenso
5 ☐ Horrible
6 ☐ Insufrible

PRODUCTIVIDAD

En los últimas 3 meses, ¿cuántos días ha dejado de trabajar o de realizar sus actividades cotidianas debido a su dolor?

Nº de días: |__|__|

En los últimos 3 meses, ¿cuántos días ha tenido que acudir a su trabajo o ha realizado sus actividades cotidianas con síntomas u otros problemas relacionados con su dolor?

Nº de días: |__|__|

En relación a la pregunta anterior, ¿cómo describiría su rendimiento promedio en el trabajo o en sus actividades cotidianas en esos días?

0%	10%	20%	30%	40%	50%	60%	70%	80%	90%	100%
☐	☐	☐	☐	☐	☐	☐	☐	☐	☐	☐

Rendimiento nulo — Pleno rendimiento

Pfizer

A0081109 - Estudio LIDO

Iniciales: ☐☐☐

Nº PACIENTE ☐

Visita Final (Semana 12)

Form 8 - FINALIZACIÓN DEL ESTUDIO

ESTADO FINAL DEL PACIENTE

MARQUE SOLAMENTE UNA DE LAS SIGUIENTES:

1 ☐ El paciente completó el estudio

SI NO COMPLETÓ, indique el MOTIVO PRINCIPAL DE LA RETIRADA:

2 ☐ Evento(s) Adverso(s)

3 ☐ No cumplió los criterios de selección

4 ☐ Pérdida de seguimiento

5 ☐ A petición del paciente

6 ☐ El paciente falleció

7 ☐ Otro. Especificar: ____________________

CUESTIONARIOS PARA EL PACIENTE

Recuerde que el paciente debe rellenar en ESTA VISITA los cuestionarios de las páginas 12-15. Por favor, revise que los ha completado correctamente

REGISTRO SEMANAL

No olvide recoger el REGISTRO SEMANAL DEL DOLOR entregado al paciente en la visita anterior

A0081109 - Estudio LIDO

Iniciales: | | |

Nº PACIENTE

Visita Final (Semana 12)

Form 5 - CUESTIONARIOS PARA EL PACIENTE (1 de 4)

CUESTIONARIO DE INCAPACIDAD DE SHEEHAN

Señale con una cruz en el número que describa mejor su situación actual.

A causa de sus síntomas su trabajo se ha visto perjudicado

No, en absoluto — Levemente — Moderadamente — Marcadamente — Extremadamente

☐ 0 ☐ 1 ☐ 2 ☐ 3 ☐ 4 ☐ 5 ☐ 6 ☐ 7 ☐ 8 ☐ 9 ☐ 10

A causa de sus síntomas su vida social y sus actividades de tiempo libre se han visto perjudicadas

No, en absoluto — Levemente — Moderadamente — Marcadamente — Extremadamente

☐ 0 ☐ 1 ☐ 2 ☐ 3 ☐ 4 ☐ 5 ☐ 6 ☐ 7 ☐ 8 ☐ 9 ☐ 10

A causa de sus síntomas su vida familiar y sus responsabilidades domésticas se han visto perjudicadas

No, en absoluto — Levemente — Moderadamente — Marcadamente — Extremadamente

☐ 0 ☐ 1 ☐ 2 ☐ 3 ☐ 4 ☐ 5 ☐ 6 ☐ 7 ☐ 8 ☐ 9 ☐ 10

ESTRÉS PERCIBIDO

Desde su última visita, ¿cuánto le han dificultado la vida los eventos estresantes y los problemas personales como los del trabajo, la casa, de salud o económicos?

No, en absoluto — Levemente — Moderadamente — Marcadamente — Extremadamente

☐ 0 ☐ 1 ☐ 2 ☐ 3 ☐ 4 ☐ 5 ☐ 6 ☐ 7 ☐ 8 ☐ 9 ☐ 10

APOYO SOCIAL PERCIBIDO

Durante la última semana, ¿qué porcentaje de apoyo ha recibido de amigos, familiares, compañeros de trabajo, etc., respecto al apoyo que ha necesitado?

Ningún apoyo en absoluto — Un poco — Moderado — Considerable — El apoyo ideal

☐ 0% ☐ 10 ☐ 20 ☐ 30 ☐ 40 ☐ 50 ☐ 60 ☐ 70 ☐ 80 ☐ 90 ☐ 100%

A0081109 - Estudio LIDO

Iniciales: Nº PACIENTE

Visita Final (Semana 12)

Form 5 - CUESTIONARIOS PARA EL PACIENTE (2 de 4)

ESCALA HOSPITALARIA DE DEPRESIÓN Y ANSIEDAD (HAD)

Los médicos son conscientes de que las emociones juegan un papel importante en la mayoría de las enfermedades. Si su médico se informa de estos estado de ánimo podrá ayudarle mejor.

Este cuestionario se ha diseñado para ayudar a que su médico sepa cómo se siente usted. No tome en cuenta los números impresos al margen del cuestionario. Lea cada sección y marque con una cruz la respuesta que más se ajuste a su estado de ánimo durante la última semana. No piense mucho las respuestas. Probablemente una reacción espontánea ante cada pregunta refleje mejor sus verdaderos sentimientos que una respuesta meditada durante mucho tiempo.

1. Me siento tenso/a o "nervioso/a":

3 ☐ La mayor parte del tiempo 2 ☐ Muchas veces 1 ☐ De vez en cuando, de repente 0 ☐ Nunca

2. Todavía disfruto con lo que antes me gustaba:

0 ☐ Como siempre 1 ☐ Un poco menos 2 ☐ Sólo un poco 3 ☐ Casi nada

3. Me viene una sensación de miedo, como si algo terrible me fuera a suceder:

3 ☐ Definitivamente y es muy fuerte 2 ☐ Sí, pero no es muy fuerte 1 ☐ Un poco, pero me preocupa 0 ☐ Nada

4. Puedo reírme y me hace gracia el aspecto divertido de las cosas:

0 ☐ Al igual que siempre lo hacía 1 ☐ Un poco menos ahora 2 ☐ Categóricamente creo que menos 3 ☐ Nunca

5. Tengo la mente llena de preocupaciones:

3 ☐ Gran parte del tiempo 2 ☐ Con bastante frecuencia 1 ☐ A veces, aunque no muy a menudo 0 ☐ Sólo en ocasiones

6. Me siento alegre:

3 ☐ Nunca 2 ☐ No muy a menudo 1 ☐ A veces 0 ☐ Casi siempre

7. Puedo descansar y relajarme:

0 ☐ Sí, no tengo ningún problema al respecto 1 ☐ Por lo general 2 ☐ No muy a menudo 3 ☐ Nunca

8. Me da la impresión que me demoro más que antes en hacer las cosas:

3 ☐ Prácticamente en todo momento 2 ☐ Muy a menudo 1 ☐ A veces 0 ☐ Nunca

9. Me viene una sensación de miedo, como un vacío en el estómago:

0 ☐ Nunca 1 ☐ En ciertas ocasiones 2 ☐ Con bastante frecuencia 3 ☐ Muy a menudo

10. He perdido interés en mi aspecto físico:

3 ☐ Categóricamente, sí 2 ☐ No me preocupa tanto como debiera 1 ☐ Quizá no me preocupe tanto como antes 0 ☐ Me preocupo al igual que siempre

11. Me siento inquieto/a, como si necesitara estar en constante movimento:

3 ☐ Mucho 2 ☐ Bastante 1 ☐ No mucho 0 ☐ Nada

12. Me siento optimista respecto a las cosas que están por venir:

0 ☐ Igual que siempre 1 ☐ Menos de lo que acostumbraba 2 ☐ Mucho menos de lo que acostumbraba 3 ☐ Casi nada

13. Me asaltan sentimientos repentinos de pánico:

3 ☐ Con muchísima frecuencia 2 ☐ Bastante a menudo 1 ☐ No muy a menudo 0 ☐ Nunca

14. Me divierto con un buen libro, la radio, o un programa de televisión:

0 ☐ A menudo 1 ☐ A veces 2 ☐ No muy a menudo 3 ☐ Rara vez

Compruebe ahora que ha contestado a todas las preguntas

A0081109 - Estudio LIDO

Iniciales: ☐☐☐

Nº PACIENTE ☐

Visita Final (Semana 12)

Form 5 - CUESTIONARIOS PARA EL PACIENTE (3 de 4)

CUESTIONARIO DE SALUD EuroQoL-5D (EQ-5D)

Marque con una cruz la respuesta de cada apartado que mejor describa su estado de salud *en el día de hoy*

El mejor estado de salud imaginable

Movilidad

1 ☐ No tengo problemas para caminar

2 ☐ Tengo algunos problemas para caminar

3 ☐ Tengo que estar en la cama

Cuidado personal

1 ☐ No tengo problemas con el cuidado personal

2 ☐ Tengo algunos problemas para lavarme o vestirme

3 ☐ Soy incapaz de lavarme o vestirme

Actividades cotidianas (p. ej., trabajar, estudiar, hacer las tareas domésticas, actividades familiares o durante el tiempo libre)

1 ☐ No tengo problemas para realizar mis actividades cotidianas

2 ☐ Tengo algunos problemas para realizar mis actividades cotidianas

3 ☐ Soy incapaz de realizar mis actividades cotidianas

Dolor / malestar

1 ☐ No tengo dolor ni malestar

2 ☐ Tengo moderado dolor o malestar

3 ☐ Tengo mucho dolor o malestar

SU ESTADO DE SALUD HOY

Ansiedad / depresión

1 ☐ No estoy ansioso ni deprimido

2 ☐ Estoy moderadamente ansioso o deprimido

3 ☐ Estoy muy ansioso o deprimido

Comparado con mi estado general de salud durante los últimos 12 meses, mi estado de salud hoy es:
(POR FAVOR, PONGA UNA CRUZ EN EL CUADRO)

1 ☐ Mejor

2 ☐ Igual

3 ☐ Peor

Para ayudar a la gente a describir lo bueno y lo malo que es su estado de salud hemos dibujado una escala parecida a un termómetro en la cual se marca con un 100 el mejor estado de salud que se pueda imaginar y con un 0 el peor estado de salud que se pueda imaginar.

Nos gustaría que nos indicara en esta escala, en su opinión, lo bueno o malo que es su estado de salud en el día de *hoy*.

El peor estado de salud imaginable

A0081109 - Estudio LIDO

Iniciales: [ ][ ][ ]

Nº PACIENTE [ ]

Visita Final (Semana 12)

Form 5 - CUESTIONARIOS PARA EL PACIENTE (4 de 4)

MÓDULO DE SUEÑO DEL M.O.S.

SU SUEÑO

	0-15 min.	16-30 min.	31-45 min.	46-60 min.	más de 60 min.
1. ¿Cuánto tiempo ha tardado habitualmente en dormirse durante las 4 últimas semanas? (marque un solo número)	☐ 1	☐ 2	☐ 3	☐ 4	☐ 5

2. De promedio, ¿cuántas horas ha dormido **cada noche** durante **las 4 últimas semanas**?

[ ][ ] Escriba el número de horas por noche

¿Con qué frecuencia durante las 4 últimas semanas?...

(Marque un solo número por cada pregunta)

	Siempre	Casi siempre	Muchas veces	Algunas veces	Sólo alguna vez	Nunca
3. ¿ha notado que su sueño no era tranquilo (moviéndose de forma inquieta sintiéndose tenso/a, hablando, etc, mientras dormía)?	☐ 1	☐ 2	☐ 3	☐ 4	☐ 5	☐ 6
4. ¿ha dormido lo suficiente como para sentirse descansado/a al despertarse por la mañana?	☐ 1	☐ 2	☐ 3	☐ 4	☐ 5	☐ 6
5. ¿se ha despertado con falta de aire o con dolor de cabeza?	☐ 1	☐ 2	☐ 3	☐ 4	☐ 5	☐ 6
6. ¿se ha sentido soñoliento/a o dormido/a durante el día?	☐ 1	☐ 2	☐ 3	☐ 4	☐ 5	☐ 6
7. ¿ha tenido problemas para quedarse dormido/a?	☐ 1	☐ 2	☐ 3	☐ 4	☐ 5	☐ 6
8. ¿se ha despertado mientras dormía y le ha costado volverse a dormir?	☐ 1	☐ 2	☐ 3	☐ 4	☐ 5	☐ 6
9. ¿le ha costado mantenerse despierto/a durante el día?	☐ 1	☐ 2	☐ 3	☐ 4	☐ 5	☐ 6
10. ¿ha roncado mientras dormía?	☐ 1	☐ 2	☐ 3	☐ 4	☐ 5	☐ 6
11. ¿se ha echado siestas (de 5 minutos o más) durante el día?	☐ 1	☐ 2	☐ 3	☐ 4	☐ 5	☐ 6
12. ¿ha dormido todo lo que necesitaba?	☐ 1	☐ 2	☐ 3	☐ 4	☐ 5	☐ 6
